# Impact of sinus rhythm versus atrial fibrillation on left ventricular remodeling after transcatheter aortic valve replacement

**DOI:** 10.1007/s00392-021-01810-5

**Published:** 2021-02-10

**Authors:** Jakob Ledwoch, Carolin Fröhlich, Ida Olbrich, Felix Poch, Ruth Thalmann, Carmen Fellner, Christian Bradaric, Karl-Ludwig Laugwitz, Christian Kupatt, Petra Hoppmann

**Affiliations:** 1 Klinik und Poliklinik für Innere Medizin I, Klinikum rechts der isar, Technical University of Munich, Munich, Germany; 2 Klinik für Kardiologie, Pneumologie und Internistische Intensivmedizin, München Klinik Neuperlach, Munich, Germany; 3 DZHK (German Center for Cardiovascular Research), Partner Site Munich Heart Alliance, Munich, Germany

**Keywords:** TAVR, Atrial fibrillation, Remodeling, Left ventricular mass

## Abstract

**Aims:**

Atrial fibrillation (AF) is associated with increased mortality after transcatheter aortic valve replacement (TAVR). Cerebrovascular complications and bleeding events associated with anticoagulation therapy are discussed to be possible causes for this increased mortality. The present study sought to assess whether AF is associated with impaired left ventricular (LV) reverse remodeling representing another possible mechanism for poor outcome.

**Methods:**

All patients who underwent TAVR in our institution and had 1-year echocardiography follow-up were included. LV mass index (LVMI) at baseline and follow-up as well as LVMI change at 1 year were assessed with respect to the presence of AF (either at baseline or during hospitalization after TAVR) and sinus rhythm (SR).

**Results:**

A total of 213 patients (*n* = 95 in AF; *n* = 118 in SR) were enrolled in the present study. Patients with AF had higher LVMI at 1 year compared to those with SR (173 ± 61 g/m^2^ vs. 154 ± 55 g/m^2^; *p* = 0.02) and they showed lower relative LVMI change at 1 year (− 2 ± 28% vs. − 9 ± 29%; *p* = 0.04). In linear regression analysis, AF was independently associated with relative LVMI change (regression coefficient *ß* 0.076 [95% CI 0.001–0.150]; *p* = 0.04). With respect to clinical outcome depending on AF and LVMI regression, the Kaplan–Meier estimated event-free of death or cardiac rehospitalization at 3 years was lowest among patients with AF and no LVMI regression.

**Conclusions:**

The present study identified a significant association of AF with changes in LVMI after TAVR, which was also shown to be associated with clinical outcome.

**Supplementary Information:**

The online version contains supplementary material available at 10.1007/s00392-021-01810-5.

## Introduction

Atrial fibrillation (AF) is one of the most prevalent comorbidities in patients undergoing transcatheter aortic valve replacement (TAVR) [1–3]. Mostly, AF is already present prior to TAVR but it can also occur after the procedure [2, 4, 5]. In high-risk patients, the prevalence can reach 50% when combining pre-existing and new-onset post-procedural AF. There is large evidence that patients with AF show a worse prognosis compared to those with sinus rhythm (SR) after TAVR [2, 4–8]. The reason for this are cerebrovascular complications and bleeding events associated with oral anticoagulation therapy [7] beside the more progressive heart disease observed in AF. Apart from cerebrovascular and bleeding complications, AF is also known to be associated with negative left ventricular (LV) remodeling outside of TAVR populations [9]. Mechanisms for that include loss of atrioventricular synchrony leading to impaired ventricular filling and increased myocardial fibrosis [9, 10].

There is a growing body of evidence that positive LV remodeling expressed by LV mass regression after TAVR is associated with increased survival [11, 12]. However, the association of LV mass alterations following TAVR with AF as another possible factor influencing the outcome after the procedure is unknown. Therefore, the present study sought to evaluate the impact of AF on changes of LV mass index (LVMI) and its association with outcome in patients after TAVR.

## Methods

### Patient population

Patients with severe symptomatic AS undergoing TAVR in our institution were consecutively included in a single-center prospective observational TAVR study since January 2015. Each patient provided written informed consent. The study was approved by the hospital’s ethics committee (number of the ethics committee approval: 314/16s) and performed according to the Decleration of Helsinki. For the purpose of the present analysis, patients were assigned into two groups according to the presence of AF (paroxysmal, persistent or permanent) or SR.

### AF detection

Patients were assigned to the AF group if they had previously known AF or atrial flutter at baseline or a new diagnosis of AF or atrial flutter during the index hospitalization. For the detection of AF or atrial flutter during the index hospitalization patients received at least one 12-lead EKG pre-procedurally, one 12-lead EKG directly after the TAVR procedure and one 12-lead EKG prior to discharge. In addition, patients were on continuous EKG monitoring after the TAVR procedure for at least 24 h. AF therapy (rhythm versus rate control) was at the discretion of the treating physician based on the patient´s clinical symptoms, left atrial size, comorbidities and age.

### Echocardiography

Echocardiographic assessment was obtained at baseline and 12 months after TAVR.

All patients received an echocardiographic exam following the current guidelines for the management of aortic valve disease [13, 14]. Valvular disease severity including the severity of paravalvular aortic regurgitation after TAVR was graded on a three-stage scale from mild to severe according to current guidelines [14–16]. LV function was assessed by biplane measures in two and four-chamber view using the Simpson biplane formula. LV mass was calculated according to the American and European Society of Echocardiography [17]: 0.8 × (1.04((LVEDD × LV posterior wall × LV septal wall)^3^ × (LVEDD)^3^)) + 0.6 g. LV mass was indexed to the body surface area [17]. Guideline recommended definition of left ventricular hypertrophy (LVH) severity was used as follows [18]: no LVH (< 96 g/m^2^ for women, < 116 g/m^2^ for men); mild LVH (96–108 g/m^2^ for women, 116–131 g/m^2^ for men); moderate LVH (109–121 g/m^2^ for women, 132–148 g/m^2^ for men); severe LVH (> 121 g/m^2^ for women, > 148 g/m^2^ for men). LVMI regression was defined as a decrease of LVMI at follow-up compared to baseline.

### Procedure

General decision for TAVR was made by an interdisciplinary heart team consisting of cardiologists and cardiac surgeons. TAVR was performed in general anesthesia under transoesophageal echocardiography and fluoroscopy guidance. The transfemoral approach was used as the default access route. In most patients either Evolut R (Medtronic, Minneapolis, USA) or Sapien S3 (Edwards, Irvine, USA) prosthetic valves were implanted.

### Endpoint definitions

Clinical follow-up including assessment of symptoms and adverse events was performed at 4 weeks, 3 months, 6 months, 12 months after TAVR and yearly thereafter. Patients who were not available for clinical follow-up were contacted by phone. All clinical events were systematically collected and evaluated by our internal TAVR board. Complications were defined according to the Valve Academic Research Consortium-2 criteria [19]. Primary outcome measures were LVMI at follow-up and change of LVMI between baseline and 1-year follow-up according to the presence of AF compared to SR. Secondary outcome measures were changes in LVEDD and LV-EF, the prevalence of AF in different LV severity classes at follow-up and clinical outcome including the composite of death or rehospitalization for heart failure.

### Statistical methods

Continuous variables were reported as mean with standard deviation or as median with interquartile range (IQR). Categorical variables were expressed as numbers and percentages. Between-group comparisons were performed using student’s *t* test for normally distributed data. For non-normally distributed data Mann–Whitney *U* test was used. Categorial data were analyzed by Chi-Square or exact Fisher test. Paired data analysis was performed by paired *t* test or Wilcoxon test. The association of AF with relative LVMI change during follow-up was assessed using multivariable linear regression. All variables showing a *p* value < 0.10 at univariable analysis were entered into this model. Mortality and rehospitalization for heart failure were calculated by means of Kaplan–Meier method with log-rank testing. A total of four groups (SR with LVMI regression vs. SR without LVMI regression vs. AF with LVMI regression vs. AF without LVMI regression) were compared. A *p* value < 0.05 was considered significant. Statistical analyses were performed using SPSS, version 26 (IBM, Chicago, USA).

## Results

### Baseline characteristics

Between January 2015 and February 2020, 367 patients underwent TAVR in our institution. Of them, a total of 213 patients with complete echocardiographic follow-up at 1 year were included in the present analysis. AF was diagnosed in 95 patients (45%) with AF prior to TAVR in 88 patients and new AF after TAVR in seven patients. Of these 95 patients, 20 patients had paroxysmal AF, 18 patients had persistent AF and 57 patients had permanent AF. New AF between hospital discharge and 1-year follow-up was not diagnosed. A total of five patients received amiodarone as rhythm control therapy. Cardioversion was performed in four out of the seven patients with new AF after the procedure.

**Fig. 1:**
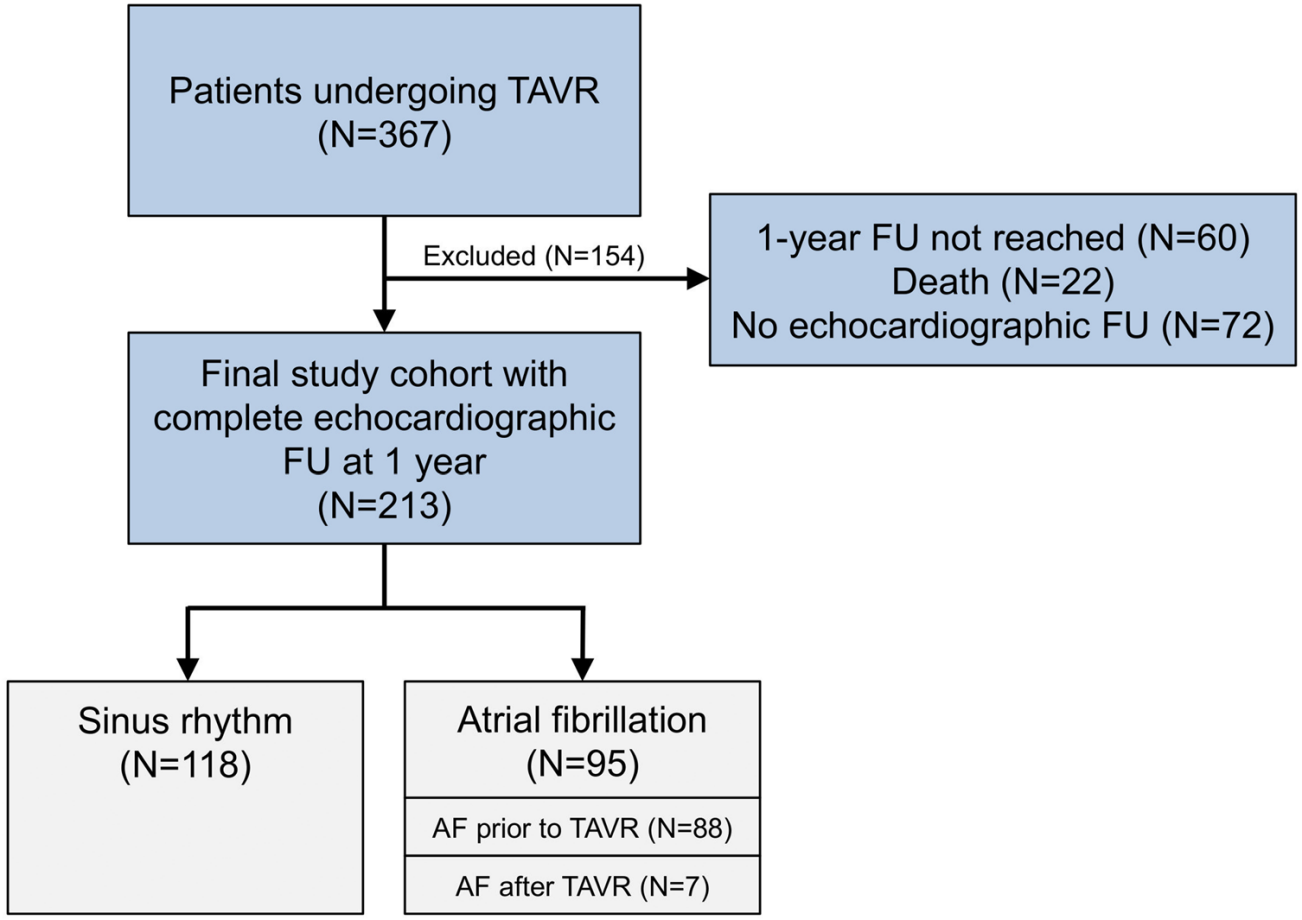
Study flow chart

The study flow is illustrated in Fig. 1. Baseline characteristics of patients with AF compared to those with SR are presented in Table 1. Patients with AF were slightly older (82 ± 7 vs. 79 ± 8 years; *p* = 0.02) and suffered more often from chronic renal failure (57% vs. 39%; *p* = 0.02). Baseline echocardiography showed differences in left atrial dimension, right ventricular dimension and function as well as tricuspid regurgitation between AF and SR (Table 2). With respect to procedural details, no significant differences were found with regard to vascular access, prosthesis type and prosthesis size (Table 3). Hospital complications and prosthesis function were similar in both groups (Table 3).

**Table 1:** Baseline characteristics

	Atrial fibrillation (*N* = 95)	Sinus rhythm (*N* = 118)	*p* value
Age (years)	82 ± 7	79 ± 8	0.02
Female	50% (47)	39% (46)	0.13
Body mass index (kg/m^2^)	26.3 ± 5.6	25.7 ± 4.6	0.44
Arterial hypertension	88% (84)	90% (106)	0.53
Dyslipidemia	59% (56)	66% (78)	0.33
Smoker	15% (14)	17% (20)	0.66
Diabetes mellitus	26% (25)	22% (26)	0.40
Coronary artery disease	67% (64)	68% (80)	0.95
Previous PCI	44% (42)	48% (56)	0.60
Previous CABG	5% (5)	7% (8)	0.65
Previous stroke	14% (13)	7% (8)	0.09
PAOD	18% (17)	17% (20)	0.86
Carotid artery disease	5% (5)	9% (11)	0.26
COPD	17% (16)	9% (11)	0.10
Chronic renal failure (eGFR < 60 ml/min)	57% (54)	39% (46)	0.02
Pacemaker	15% (14)	11% (13)	0.42
ICD	3% (3)	2% (2)	0.48
RBBB	6% (6)	11% (13)	0.23
LBBB	8% (8)	7% (8)	0.65
EuroSCORE II	5.8 ± 4.8	4.6 ± 3.6	0.06
NYHA ≥ III	74% (70)	63% (74)	0.90
Medication			
ACE inhibitor/ARB	78% (74)	77% (91)	0.89
Betablocker	83% (79)	67% (79)	0.007
MRA	13% (12)	18% (21)	0.30

*PCI* percutaneous coronary intervention, *CABG* coronary artery bypass graft, *PAOD* peripheral arterial occlusive disease, *COPD* chronic obstructive pulmonary disease, *eGFR* estimated glomerular filtration rate, *ICD* implantable cardioverter defibrillator, *RBBB* right bundle branch block, *LBBB* left bund branch block, *ACE* angiotensin-converting enzyme, *ARB* angiotensin II receptor blocker, *MRA* mineralocorticoid receptor antagonist

**Table 2:** Echocardiographic findings

	Atrial fibrillation (*N* = 95)	Sinus rhythm (*N* = 118)	*p* value
Baseline			
LVMI (g/m^2^)	182 ± 62	176 ± 61	0.45
Severe LVH	79% (74)	78% (92)	0.89
LVEDD (mm)	46 ± 8	46 ± 7	0.86
LVESD (mm)	33 ± 10	33 ± 6	0.80
LV septal wall (mm)	14 ± 2	14 ± 2	0.92
LV posterior wall (mm)	13 ± 2	12 ± 2	0.42
LV-EF (%)	53 ± 11	53 ± 9	0.68
LA diameter (mm)	48 ± 11	43 ± 7	0.004
AV area (cm^2^)	0.9 ± 0.2	0.8 ± 0.2	0.28
AV mean gradient (mmHg)	41 ± 13	45 ± 14	0.02
TAPSE (mm)	20 ± 4	22 ± 5	0.003
RV diameter (mm)	33 ± 4	31 ± 4	0.02
RV-RA gradient (mmHg)	40 ± 13	39 ± 15	0.70
Mitral regurgitation ≥ moderate (%)	21	12	0.07
Tricuspid regurgitation ≥ moderate (%)	16	2	< 0.001
Follow-up			
LVMI (g/m^2^)	173 ± 61	154 ± 55	0.02
Severe LVH	74% (70)	55% (65)	0.005
Absolute change in LVMI (g/m^2^)	− 10 ± 54	− 22 ± 52	0.12
Relative change in LVMI (%)	− 2 ± 28	− 9 ± 29	0.04
LVEDD (mm)	47 ± 8	44 ± 6	0.003
LVESD (mm)	33 ± 8	31 ± 6	0.07
LV septal wall (mm)	13 ± 2	13 ± 2	0.23
LV posterior wall (mm)	12 ± 2	12 ± 2	0.79
LV-EF (%)	52 ± 11	56 ± 7	0.004
LA diameter (mm)	48 ± 12	44 ± 28	0.01
AV area (cm^2^)	1.8 ± 0.4	1.7 ± 0.3	0.29
AV mean gradient (mmHg)	10 ± 4	10 ± 3	0.84
Aortic regurgitation ≥ moderate (%)	0	0	–
TAPSE (mm)	19 ± 4	21 ± 4	0.001
RV diameter (mm)	33 ± 5	31 ± 5	0.02
RV-RA gradient (mmHg)	37 ± 11	34 ± 12	0.22
Mitral regurgitation ≥ moderate (%)	21	6	0.001
Tricuspid regurgitation ≥ moderate (%)	23	3	< 0.001

*LVMI* left ventricular mass index, *LVH* left ventricular hypertrophy, *LVEDD* left ventricular end-diastolic diameter, *LVESD* left ventricular endsystolic diameter, *LV-EF* left ventricular ejection fraction, *LA* left atrial, *AV* aortic valve, *TAPSE* tricuspid annular plane systolic excursion, *RV* right ventricular, *RA* right atrial

**Table 3:** Procedural details and hospital outcome

	Atrial fibrillation (*N* = 95)	Sinus rhythm (*N* = 118)	*p* value
Procedural details			
Transfemoral access	89% (105)	86% (81)	0.54
Prosthesis type			
Sapien S3	75% (72)	81% (96)	0.59
Evolut R	19% (18)	15% (18)	
Other	5% (5)	3% (4)	
Prosthesis size (mm)	25.6 ± 2.4	26.2 ± 3.0	0.10
Hospital outcome			
Myocardial infarction	1% (1)	0% (0)	0.44
Stroke	1% (1)	1% (1)	1.00
Minor vascular complications	13% (12)	13% (15)	0.97
Major vascular complications	3% (3)	2% (2)	0.66
Minor bleeding	8% (7)	5% (6)	0.57
Major bleeding	4% (4)	3% (3)	0.70
Life-threatening bleeding	2% (2)	1% (1)	0.58
Acute kidney injury stage 1	1% (1)	1% (1)	1.00
Acute kidney injury stage 2	1% (1)	1% (1)	1.00
Acute kidney injury stage 3	3% (3)	3% (4)	1.00
New RBBB	2% (2)	0% (0)	0.09
New LBBB	8% (7)	3% (3)	0.09
New pacemaker	11% (10)	9% (11)	0.73
AV area (cm^2^)	1.70 ± 0.39	1.71 ± 0.41	0.26
AV mean gradient (mmHg)	10.2 ± 3.5	9.9 ± 4.6	0.21
Paravalvular AR ≥ moderate	0% (0)	0% (0)	–

*RBBB* right bundle branch block, *LBBB* left bund branch block, *AV* aortic valve, *AR* aortic regurgitation

### Cardiac remodeling

**Fig. 2:**
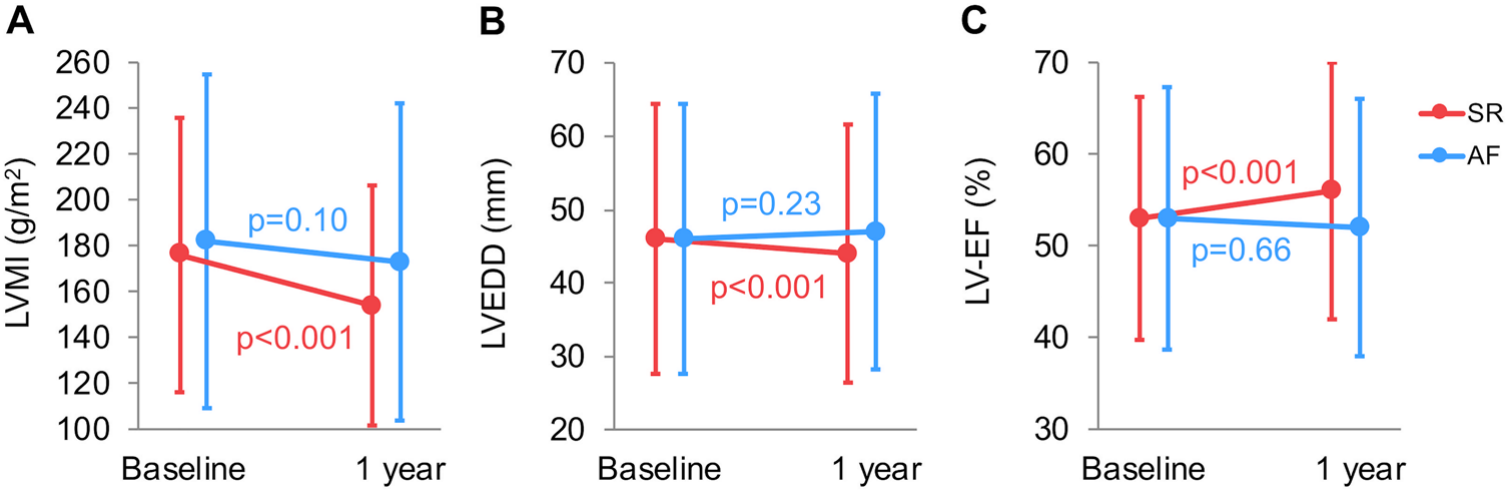
Changes in LV remodeling parameters in patients with AF versus SR. In contrast to AF, patients in SR experienced a significant reduction in LVMI (**a**), reduction in LVEDD (**b**) and increase in LV-EF (**c**)

**Fig. 3:**
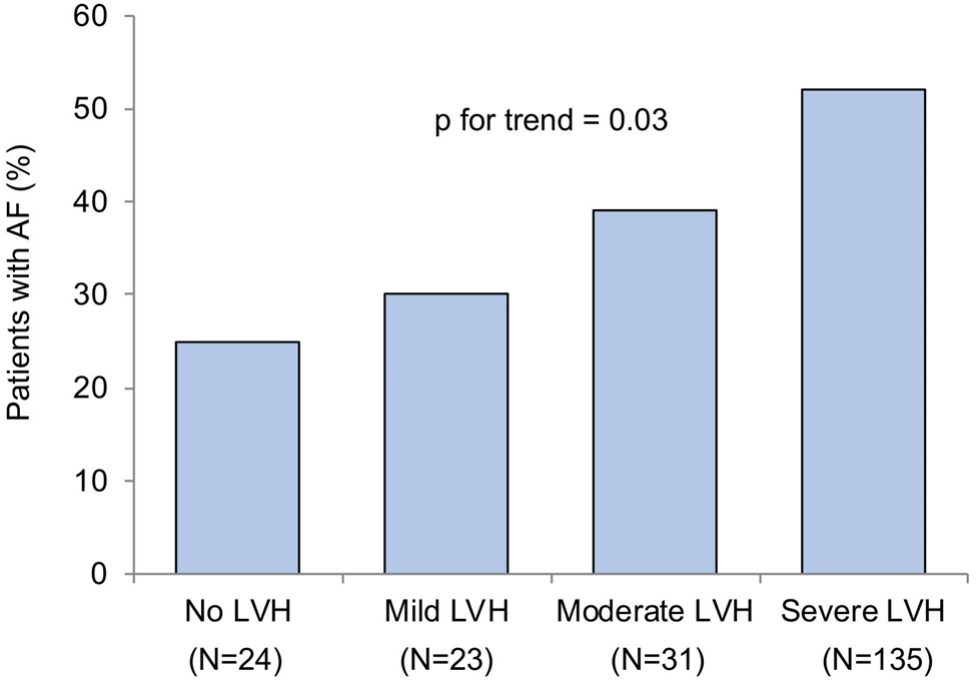
Association of AF prevalence in different classes of LVH severity at 1-year follow-up. The proportion of AF in various groups of LVH severity showed increasing AF prevalence in higher classes of LVH severity

At baseline, LVMI was similar between patients with AF and those with SR. Paired analysis showed a significant reduction in LVMI (Fig. 2a) and in LVEDD (Fig. 2b) and a significant increase in LV-EF (Fig. 2c) between baseline and 1-year follow-up in patients with SR. No significant changes in these parameters were observed in the group with AF. Relative LVMI reduction between baseline and follow-up was lower in patients with AF compared to those with SR (− 2 ± 28 vs. − 9 ± 29%; *p* = 0.04). Consequently, LVMI index was significantly higher in the AF group compared to the SR group (173 ± 61 vs. 154 ± 55 g/m^2^; *p* = 0.02) after 1 year following TAVR (Table 2). Changes of LV mass parameters dependent on different types of AF (paroxysmal, persistent or permanent) are listed in Supplementary Table S1. Whereas LV-EF at baseline was similar between both groups, it was significantly lower in patients with AF at 1 year (52 ± 11 vs. 56 ± 7%; *p* = 0.004). LVEDD was found to be significantly higher in patients with AF 1 year after TAVR (Table 2). Furthermore, the difference in more than mild mitral and tricuspid regurgitation between the two groups increased at follow-up. Figure 3 illustrates the proportion of patients with AF in different classes of LVH severity at 1-year follow-up. Rising AF prevalence was detected across increasing levels of LVH severity (*p* = 0.03). Finally, multivariable linear regression showed that AF was an independent determinant of relative change in LVMI (Table 4).

**Table 4:** Multivariable linear regression with the relative change in LVMI as the independent variable

	Regression coefficient *ß* (95% CI)	*p* value
Age	0.001 (− 0.004 to 0.006)	0.35
Female	− 0.035 (− 0.111 to 0.040)	0.36
Mitral regurgitation ≥ moderate	− 0.027 (− 0.131 to 0.077)	0.61
ACE inhibitor/ARB	− 0.058 (− 0.146 to 0.030)	0.13
Pacemaker at baseline	0.101 (− 0.010 to 0.212)	0.08
LVMI at baseline	− 0.002 (− 0.002 to − 0.001)	< 0.001
Atrial fibrillation	0.076 (0.001–0.150)	0.04

*ACE* angiotensin-converting enzyme, *ARB* angiotensin II receptor blocker, *LVMI* left ventricular mass index

### Clinical outcome

**Fig. 4:**
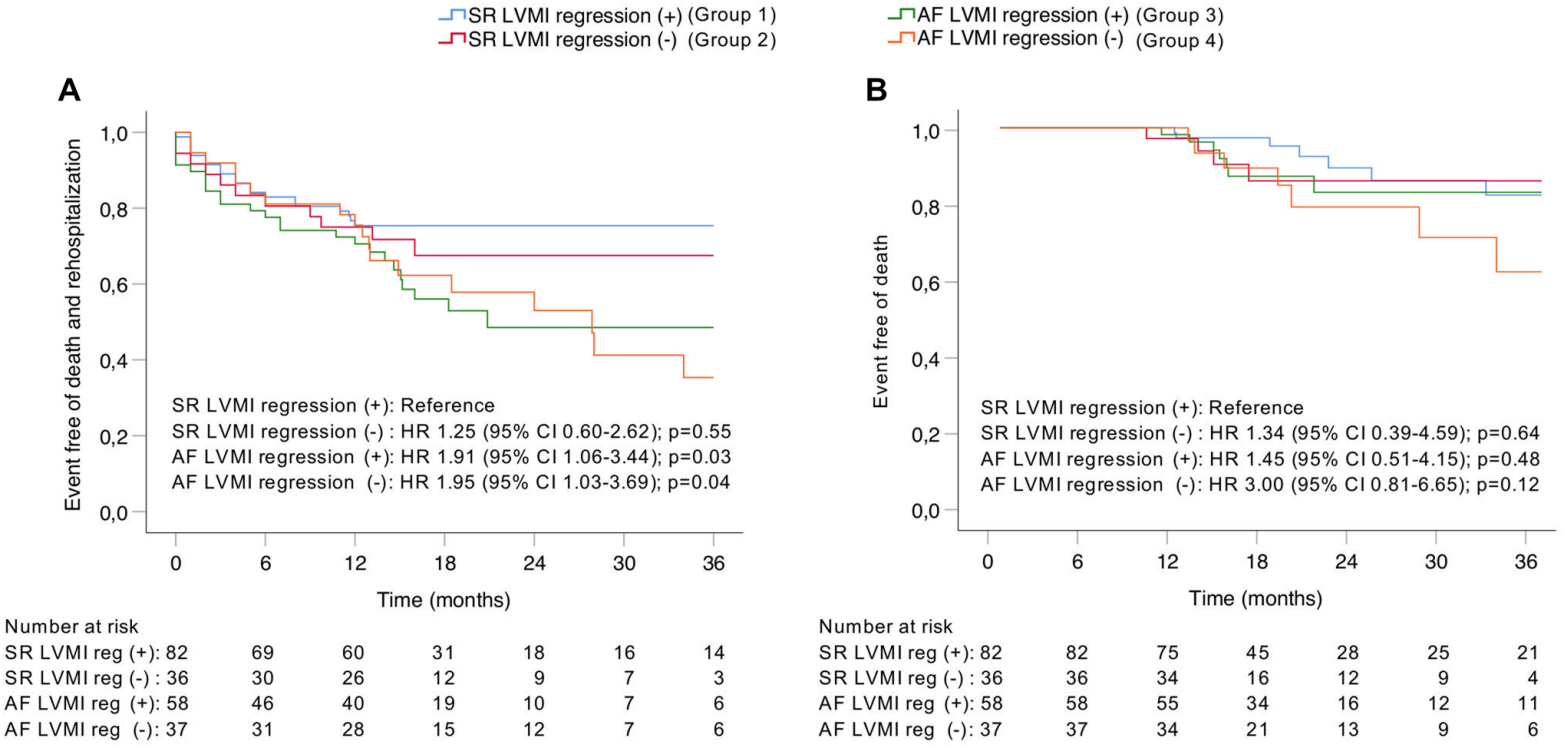
Kaplan–Meier estimated event-free of death and rehospitalization (**a**) and event-free of death (**b**) depending on cardiac rhythm and LVMI regression. A decrease of the combined endpoint of event-free of all-cause death or cardiac rehospitalization was observed across the 4 groups depending on AF or SR as well as the presence or absence of LVMI regression. The lowest rate was identified in the group with AF and absence of LV mass regression (**a**). No statistical significance was reached when only assessing mortality (**b**)

Median follow-up of the population was 18.4 months (IQR 13.7–30.4 months). A total of four groups were assessed regarding clinical outcome: SR with LVMI regression (defined as a reduction of LVMI between baseline and follow-up) (group 1); SR without LVMI regression (group 2); AF with LVMI regression (group 3); AF without LVMI regression (group 4). The Kaplan–Meier estimated event-free of death or cardiac rehospitalization at 3 years was highest in group 1 and declined across the groups. Patients in group 4 had the worst outcome (Fig. 4a). The difference in event-free of death did not reach statistical significance (Fig. 4b).

## Discussion

The present study is the first to assess the impact of AF versus SR on LV remodeling after TAVR. We were able to demonstrate a significant reduction of LVMI and LV dimensions as well as an increase in LV-EF in patients with SR 1 year after TAVR. In contrast, patients with AF did not show any significant changes in these remodeling parameters during the follow-up. This led to higher residual LVMI (173 ± 61 g/m^2^ vs. 154 ± 55 g/m^2^) and a higher proportion of severe LVH (75% vs. 55%) in the AF group. Importantly, AF was also found to be independently associated with LVMI change after TAVR. Finally, clinical outcome was assessed in different groups depending on cardiac rhythm and the presence of LVMI regression. The lowest event-free of death and cardiac rehospitalization was found in patients with AF and absence of LVMI regression. These results add new insights regarding the question why AF is associated with worse clinical outcome after TAVR.

Sub-analyses of the Placement of Aortic Transcatheter Valves (PARTNER) trials already showed a strong association of LV mass regression with clinical outcome including mortality and rehospitalization after TAVR [11, 12]. However, they did not conduct a possible influence of AF. Studies in other entities such as arterial hypertension identified an association of AF with regression of LVMI under antihypertensive medical treatment [20, 21]. Hennersdorf et al. found that patients with LVMI regression showed an AF prevalence of 2% compared to 17% in patients with LVMI progression [20]. The relationship between LV remodeling and AF is a well-known “chicken-and-egg” conundrum. On the one hand, LVMI regression is assumed to reduce LV filling pressures associated with beneficial left atrial remodeling and reduction of AF burden. On the other hand, the cascade can also be the other way around and AF itself may influence the remodeling process in the LV. This hypothesis is supported by the increasing evidence of beneficial LV remodeling due to restoration of SR by catheter ablation of AF [9, 22, 23]. In the Catheter Ablation versus Standard Conventional Therapy in Patients with Left Ventricular Dysfunction and Atrial Fibrillation (CASTLE-AF) trial, catheter ablation of AF was associated with improved overall survival and reduced rehospitalization in patients with LV dysfunction [24]. Another important secondary endpoint in this study was a change in LV-EF showing an increase of 8.0% in the ablation group versus 0.2% in the control group (*p* = 0.005). Besides an increase in LV-EF, Prabhu et al. showed a reduction in LA and LV volumes in patients after catheter ablation compared to controls with medical rate control [23]. Another study also revealed a significant decrease of LVMI in patients treated by catheter ablation compared to rate control [25]. No data are available regarding the different LV remodeling capacities between patients in SR and AF after TAVR. Jin et al. showed in their small study assessing 89 patients after surgical AVR for AS or aortic regurgitation a significantly higher LVMI in non-SR compared SR during follow-up (163 ± 8 vs. 131 ± 7 g/m^2^; *p* = 0.001), which is in line with our results.

Possible explanations for the association of AF with sustained LVH include AF-induced tachycardiomopathy. Histopathological analysis revealed inflammation-mediated diameter increase of cardiomyocytes and interstitial fibrosis in patients with tachycardiomopathy [26]. Even irrespective of tachycardiomopathy, AF was found to be associated with more severe LV fibrosis compared to SR [27]. Interstitial LV fibrosis is known to play a central role regarding the remodeling capacity of the LV. After catheter ablation of patients with LV dysfunction, the increase in LV-EF was found to be significantly higher in subjects without fibrosis compared to those with fibrosis on CMR scans (+ 22% vs. + 12%; *p* = 0.007) [23]. Only recently, Puls et al. detected greater reverse LV remodeling by means of LVMI and LVEDD after TAVR in patients with less interstitial LV fibrosis detected by LV biopsy [28]. Furthermore, AF can lead to loss of atrioventricular synchrony and various cycle length in the LV. Irregular cycle length in AF is known to lead to unfavorable hemodynamics including increased LV pressure [10], which results in increased wall stress and, ultimately, increased wall thickness [29]. In this context, one can speculate that reduced residual aortic valve area and increased residual transvalvular gradients may be associated with worse remodeling after TAVR. However, neither our study nor the PARTNER sub-analysis [11, 12] observed such association. Either the differences in residual gradients are too small to have a meaningful clinical relevance or other factors such as AF or baseline LVMI have a much greater effect on remodeling than these prosthesis parameters. In the present study, AF patients had more often chronic renal failure, which is known to be associated with LV hypertrophy and fibrosis [30]. This might be another factor for the limited reverse remodeling process in those with AF.

Our study adds further insights regarding the impact of AF after TAVR beyond the already discussed stroke and bleeding risk. The present results may stimulate the development of new therapeutic strategies of AF patients after TAVR. For example, restoration of SR by catheter ablation techniques could be beneficial. While the impact on stroke and bleeding events is uncertain, there is large evidence for improved LV remodeling with such therapy as described above. Besides favorable LV remodeling, this therapy may improve clinical outcome too. A propensity-score matched analysis from McCarthy et al. showed that surgical ablation of AF concomitant with cardiac surgery including surgical AVR in almost one half of the cases was associated with significantly higher long-term survival compared to patients who did not undergo ablation. Furthermore, survival was similar between patients with ablation-treated AF and those without AF [31]. Another option might be the consequent use of renin-angiotensin system blockers in patients with AF since they were found to be associated with LV mass regression and improved clinical outcome after TAVR [32, 33].

### Limitations

First, we are not able to definitively exclude that some patients in the SR group had AF since long-term EKG was not part of the protocol and, therefore, was not routinely performed. Second, the sample size was limited possibly leading to suboptimal statistical robustness and risk of type II error. Third, LV mass and remodeling were assessed using echocardiography. Despite this is a widely used and validated method magnetic resonance imaging and cardiac tissue biopsy could have added more detailed information regarding fibrosis and cellular alterations. Fourth, detection of AF during follow-up was based on single 12-lead EKGs and no routine holter EKG monitoring was performed. Therefore, it cannot be excluded that some patients in the SR group developed AF during follow-up. Fifth, echocardiographic follow-up was limited to 12 months and clinical follow-up was limited to 18 months. A longer observation time could enable the detection of larger changes in LV remodeling and a higher number of clinical events. Finally, since patients who died before the 12 months echocardiographic follow-up were excluded a certain survival bias cannot be ruled out.

## Conclusion

The present study showed for the first time that AF has a strong impact on LV remodeling after TAVR. Patients with AF showed less LVMI regression, which was associated with an increased rate of the composite endpoint of mortality and cardiac rehospitalization.

## Supplementary Information

Below is the link to the electronic supplementary material.

Supplementary Table S1 (DOCX 14 KB)

## Abbreviations

AF: Atrial fibrillation
EF: Ejection fraction
IQR: Interquartile range
LV: Left ventricular
LVMI: Left ventricular mass index
LVEDD: Left ventricular end-diastolic diameter
LVESD: Left ventricular endsystolic diameter
LVH: Left ventricular hypertrophy
PCI: Percutaneous coronary intervention
SR: Sinus rhythm
TAVR: Transcatheter aortic valve replacement
