# Supplementary material for: Impact of sinus rhythm versus atrial fibrillation on left ventricular remodeling after transcatheter aortic valve replacement
Source: Clin Res Cardiol. 2021 Feb 10;110(5):689–98. doi: 10.1007/s00392-021-01810-5 (PMC8099831; doi:10.1007/s00392-021-01810-5)
Supplement: Supplementary file 1 — Supplementary Table S1 (DOCX 14 KB) [file 392_2021_1810_MOESM1_ESM.docx]

Supplementary Table S1:

|  | Sinus rhythm  (N=118) | Paroxysmal/persistent AF  (N=38) | Permanent  AF  (N=57) | p-value |
| --- | --- | --- | --- | --- |
| LVMI at baseline (g/m^2^) | 176 ± 61 | 194 ± 61 | 175 ± 63 | 0.75 |
| LVMI at follow-up (g/m^2^) | 154 ± 55 | 180 ± 57 | 168 ± 63 | 0.06 |
| Absolute change in LVMI (g/m^2^) | - 22 ± 52 | - 12 ± 53 | - 8 ± 54 | 0.24 |
| Relative change in LVMI (%) | - 8.7 ± 29.7 | - 3.0 ± 25.5 | - 1.1 ± 30.6 | 0.23 |
